# Supplementary figures and images for: Phylogenetic investigation and mitochondrial genome description of ten species in nine genera of Cicadellinae from China (Hemiptera: Cicadellidae)
Source: PLoS One. 2025 Aug 12;20(8):e0329906. doi: 10.1371/journal.pone.0329906 (PMC12342267; doi:10.1371/journal.pone.0329906)

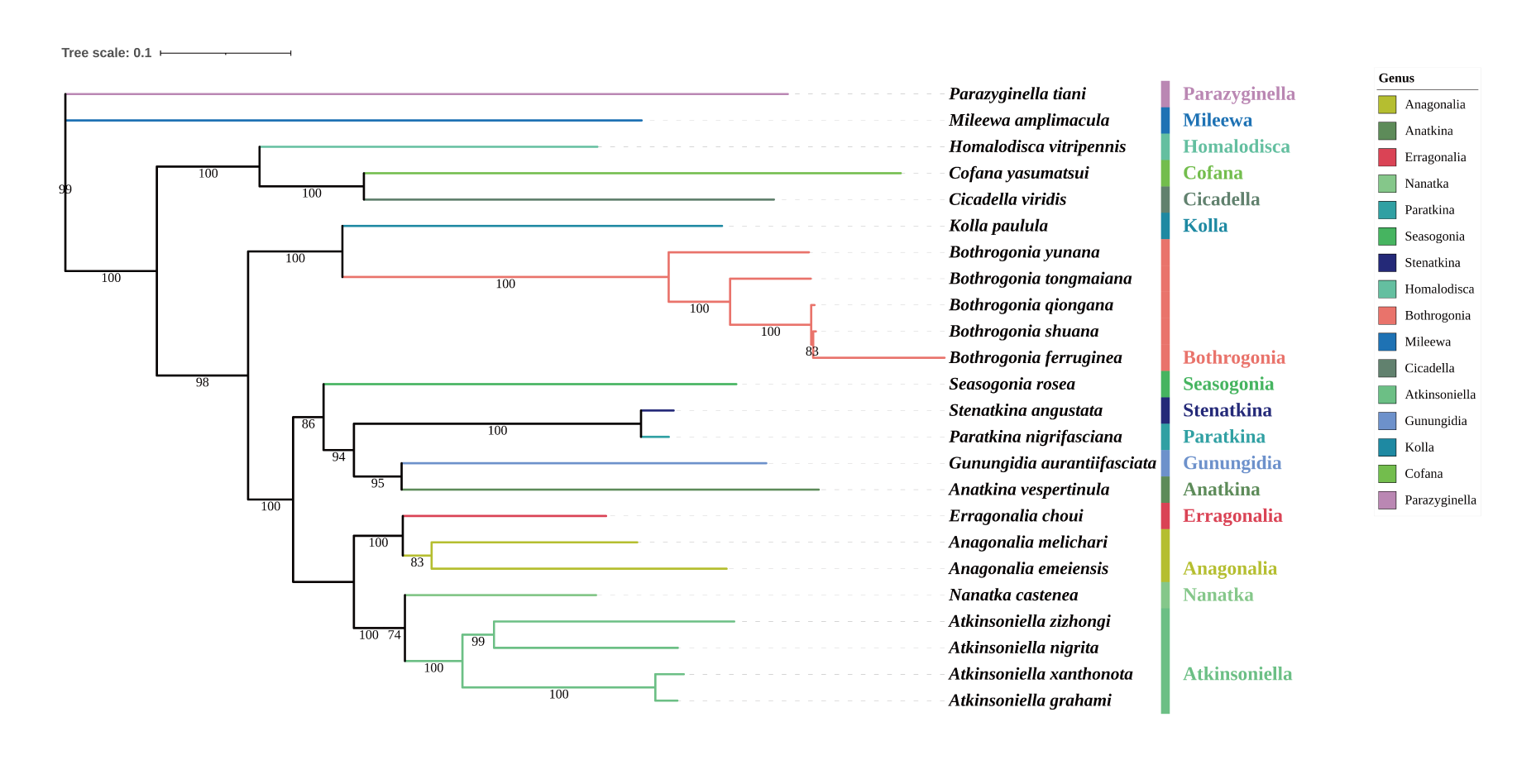

Supplement: S1 File — S1 Fig. Predicted secondary cloverleaf structure for the tRNAs of Anagonalia emeiensis. S2 Fig. Predicted secondary cloverleaf structure for the tRNAs of Anagonalia melichari. S3 Fig. Predicted secondary cloverleaf structure for the tRNAs of Anatkina vespertinula. S4 Fig. Predicted secondary cloverleaf structure for the tRNAs of Erragonalia choui. S5 Fig. Predicted secondary cloverleaf structure for the tRNAs of Gunungidia aurantiifasciata. S6 Fig. Predicted secondary cloverleaf structure for the tRNAs of kolla paulula. S7 Fig. Predicted secondary cloverleaf structure for the tRNAs of Nanatka castenea. S8 Fig. Predicted secondary cloverleaf structure for the tRNAs of Paratkina nigrifasciana. S9 Fig. Predicted secondary cloverleaf structure for the tRNAs of Seasogonia rosea. S10 Fig. Predicted secondary cloverleaf structure for the tRNAs of Stenatkina angustata. S11 Fig. Phylogenetic trees inferred by Bayesian inference (BI) based on the 13 protein-coding genes (PCGs). Bayesian posterior probabilities (BPPs) and bootstrap percentages (BP) are indicated on branches. S12 Fig. Phylogenetic trees inferred by Bayesian inference(BI) based on the 13 protein-coding genes and two rRNA genes (PCGs + rRNA). Bayesian posterior probabilities (BPPs) and bootstrap percentages (BP) are indicated. S13 Fig. Phylogenetic trees inferred by maximum likelihood (ML) based on the 13 protein-coding genes (PCGs). Bootstrap percentage (bp) is indicated on branches. S14 Fig. Phylogenetic trees inferred by maximum likelihood (ML) based on the 13 protein-coding genes and two rRNA genes (PCGs + rRNA). Bootstrap percentage (bp) is indicated on branches. S1 Table. Collection information for the 10 Cicadellidae species in this study. S2 Table. Summary statistics of the sequenced species. S3 Table. Sequence read archive accessions. S4 Table. Anagonalia emeiensis, Anagonalia melichari, Anatkina vespertinula, Erragonalia choui, Gunungidia aurantiifasciata, Kolla paulula, Nanatka castenea, Paratkina nigr [file pone.0329906.s001.zip › S14_Fig.tif]

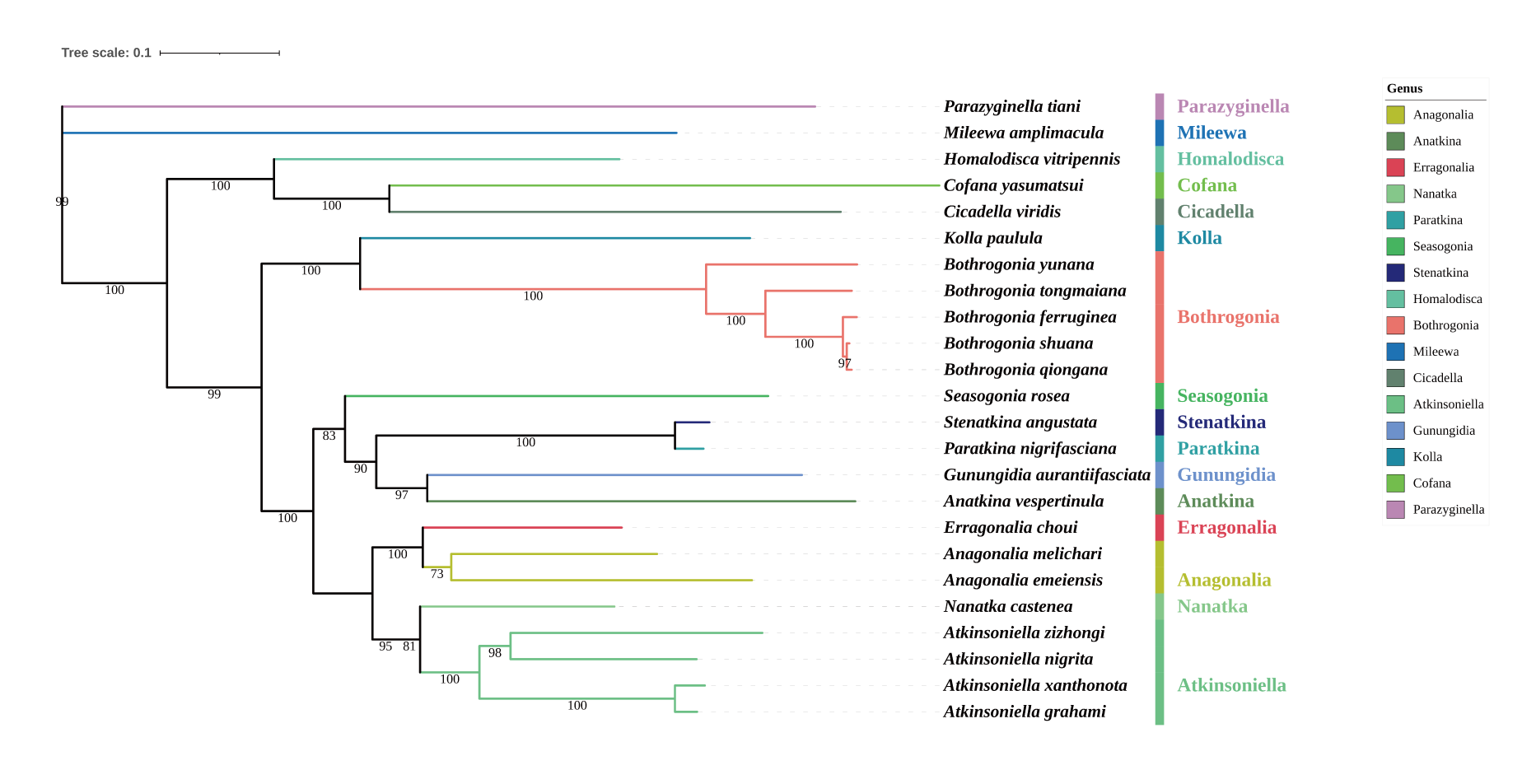

Supplement: S1 File — S1 Fig. Predicted secondary cloverleaf structure for the tRNAs of Anagonalia emeiensis. S2 Fig. Predicted secondary cloverleaf structure for the tRNAs of Anagonalia melichari. S3 Fig. Predicted secondary cloverleaf structure for the tRNAs of Anatkina vespertinula. S4 Fig. Predicted secondary cloverleaf structure for the tRNAs of Erragonalia choui. S5 Fig. Predicted secondary cloverleaf structure for the tRNAs of Gunungidia aurantiifasciata. S6 Fig. Predicted secondary cloverleaf structure for the tRNAs of kolla paulula. S7 Fig. Predicted secondary cloverleaf structure for the tRNAs of Nanatka castenea. S8 Fig. Predicted secondary cloverleaf structure for the tRNAs of Paratkina nigrifasciana. S9 Fig. Predicted secondary cloverleaf structure for the tRNAs of Seasogonia rosea. S10 Fig. Predicted secondary cloverleaf structure for the tRNAs of Stenatkina angustata. S11 Fig. Phylogenetic trees inferred by Bayesian inference (BI) based on the 13 protein-coding genes (PCGs). Bayesian posterior probabilities (BPPs) and bootstrap percentages (BP) are indicated on branches. S12 Fig. Phylogenetic trees inferred by Bayesian inference(BI) based on the 13 protein-coding genes and two rRNA genes (PCGs + rRNA). Bayesian posterior probabilities (BPPs) and bootstrap percentages (BP) are indicated. S13 Fig. Phylogenetic trees inferred by maximum likelihood (ML) based on the 13 protein-coding genes (PCGs). Bootstrap percentage (bp) is indicated on branches. S14 Fig. Phylogenetic trees inferred by maximum likelihood (ML) based on the 13 protein-coding genes and two rRNA genes (PCGs + rRNA). Bootstrap percentage (bp) is indicated on branches. S1 Table. Collection information for the 10 Cicadellidae species in this study. S2 Table. Summary statistics of the sequenced species. S3 Table. Sequence read archive accessions. S4 Table. Anagonalia emeiensis, Anagonalia melichari, Anatkina vespertinula, Erragonalia choui, Gunungidia aurantiifasciata, Kolla paulula, Nanatka castenea, Paratkina nigr [file pone.0329906.s001.zip › S13_Fig.tif]

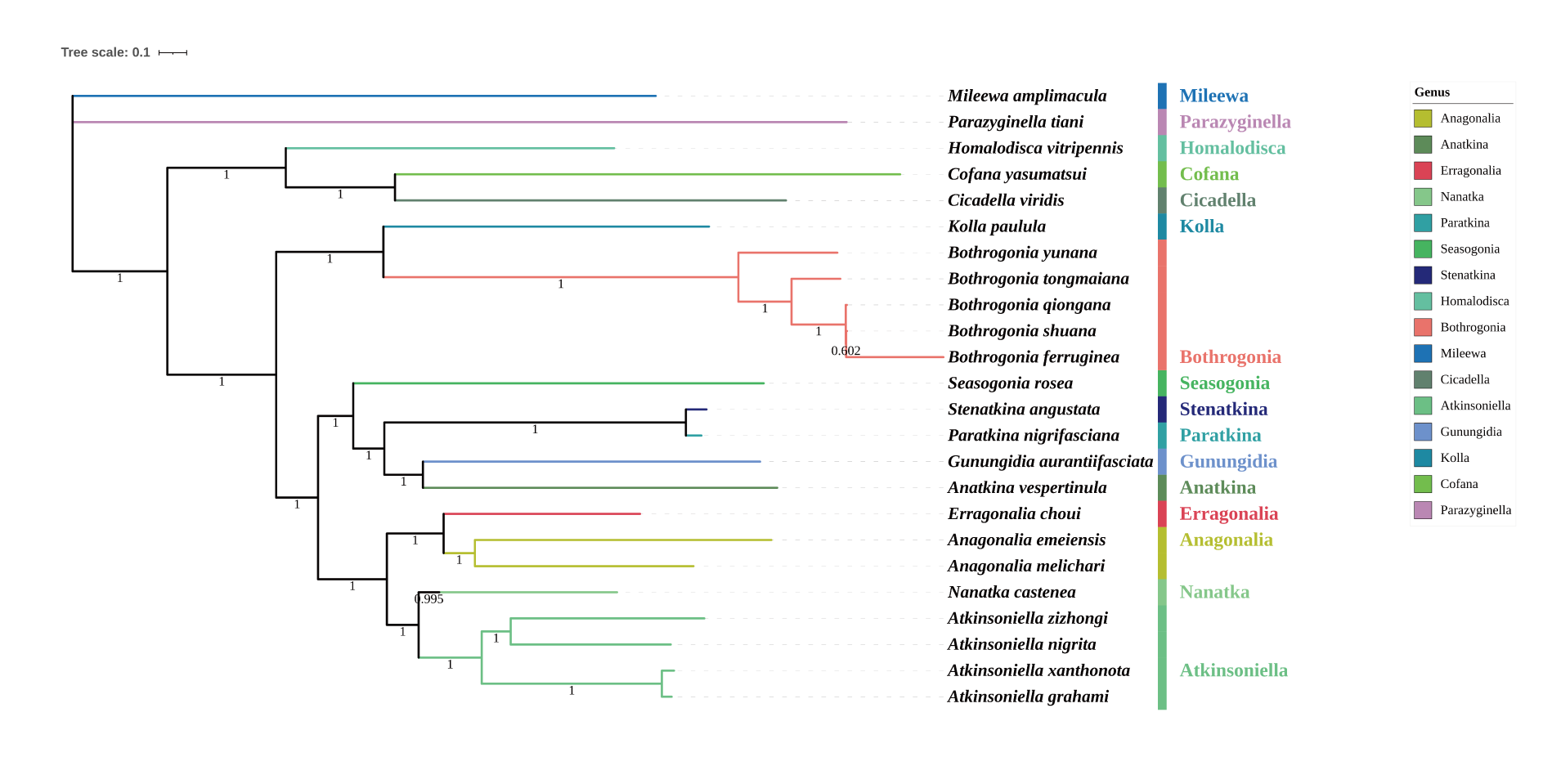

Supplement: S1 File — S1 Fig. Predicted secondary cloverleaf structure for the tRNAs of Anagonalia emeiensis. S2 Fig. Predicted secondary cloverleaf structure for the tRNAs of Anagonalia melichari. S3 Fig. Predicted secondary cloverleaf structure for the tRNAs of Anatkina vespertinula. S4 Fig. Predicted secondary cloverleaf structure for the tRNAs of Erragonalia choui. S5 Fig. Predicted secondary cloverleaf structure for the tRNAs of Gunungidia aurantiifasciata. S6 Fig. Predicted secondary cloverleaf structure for the tRNAs of kolla paulula. S7 Fig. Predicted secondary cloverleaf structure for the tRNAs of Nanatka castenea. S8 Fig. Predicted secondary cloverleaf structure for the tRNAs of Paratkina nigrifasciana. S9 Fig. Predicted secondary cloverleaf structure for the tRNAs of Seasogonia rosea. S10 Fig. Predicted secondary cloverleaf structure for the tRNAs of Stenatkina angustata. S11 Fig. Phylogenetic trees inferred by Bayesian inference (BI) based on the 13 protein-coding genes (PCGs). Bayesian posterior probabilities (BPPs) and bootstrap percentages (BP) are indicated on branches. S12 Fig. Phylogenetic trees inferred by Bayesian inference(BI) based on the 13 protein-coding genes and two rRNA genes (PCGs + rRNA). Bayesian posterior probabilities (BPPs) and bootstrap percentages (BP) are indicated. S13 Fig. Phylogenetic trees inferred by maximum likelihood (ML) based on the 13 protein-coding genes (PCGs). Bootstrap percentage (bp) is indicated on branches. S14 Fig. Phylogenetic trees inferred by maximum likelihood (ML) based on the 13 protein-coding genes and two rRNA genes (PCGs + rRNA). Bootstrap percentage (bp) is indicated on branches. S1 Table. Collection information for the 10 Cicadellidae species in this study. S2 Table. Summary statistics of the sequenced species. S3 Table. Sequence read archive accessions. S4 Table. Anagonalia emeiensis, Anagonalia melichari, Anatkina vespertinula, Erragonalia choui, Gunungidia aurantiifasciata, Kolla paulula, Nanatka castenea, Paratkina nigr [file pone.0329906.s001.zip › S12_Fig.tif]

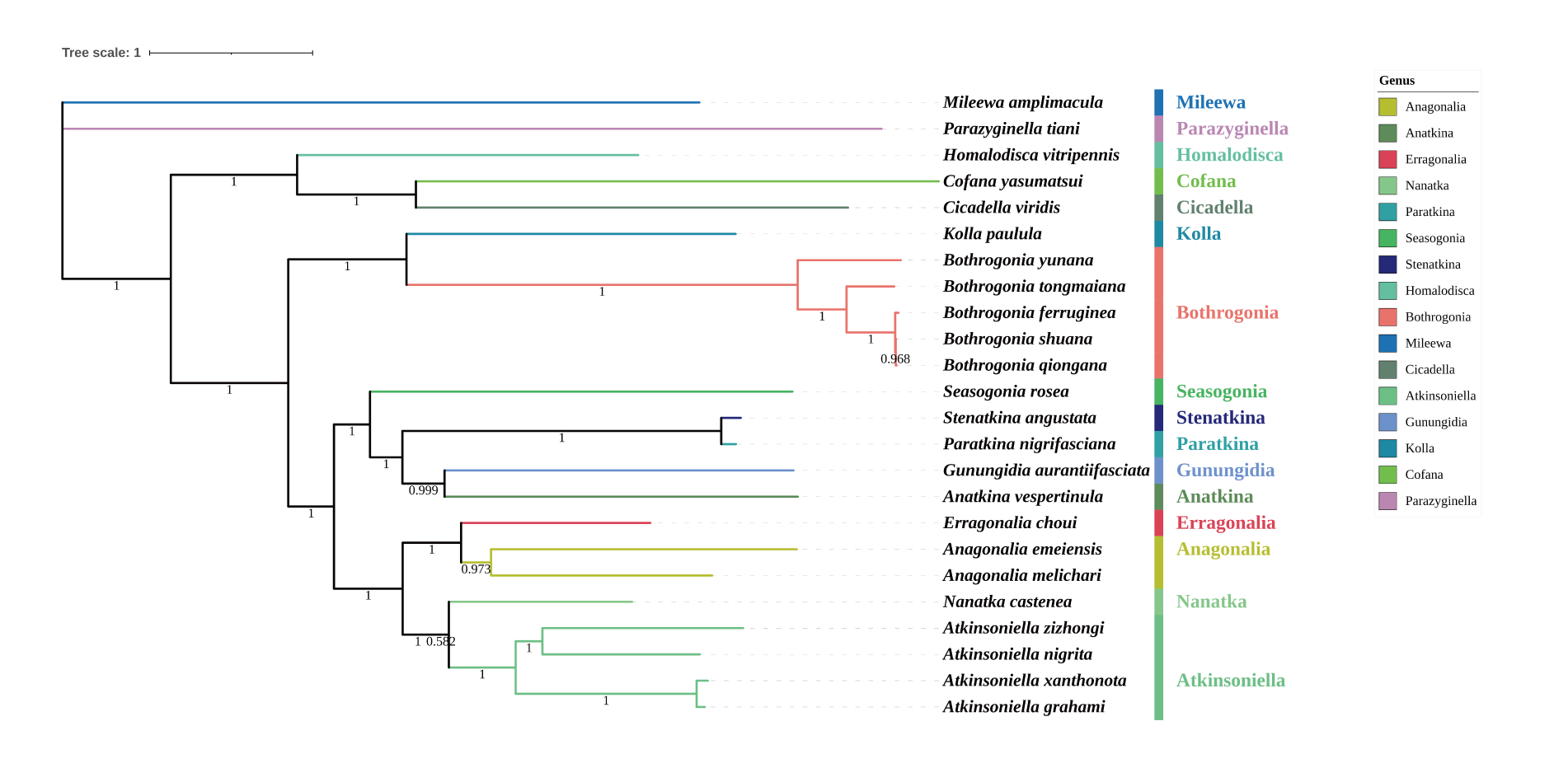

Supplement: S1 File — S1 Fig. Predicted secondary cloverleaf structure for the tRNAs of Anagonalia emeiensis. S2 Fig. Predicted secondary cloverleaf structure for the tRNAs of Anagonalia melichari. S3 Fig. Predicted secondary cloverleaf structure for the tRNAs of Anatkina vespertinula. S4 Fig. Predicted secondary cloverleaf structure for the tRNAs of Erragonalia choui. S5 Fig. Predicted secondary cloverleaf structure for the tRNAs of Gunungidia aurantiifasciata. S6 Fig. Predicted secondary cloverleaf structure for the tRNAs of kolla paulula. S7 Fig. Predicted secondary cloverleaf structure for the tRNAs of Nanatka castenea. S8 Fig. Predicted secondary cloverleaf structure for the tRNAs of Paratkina nigrifasciana. S9 Fig. Predicted secondary cloverleaf structure for the tRNAs of Seasogonia rosea. S10 Fig. Predicted secondary cloverleaf structure for the tRNAs of Stenatkina angustata. S11 Fig. Phylogenetic trees inferred by Bayesian inference (BI) based on the 13 protein-coding genes (PCGs). Bayesian posterior probabilities (BPPs) and bootstrap percentages (BP) are indicated on branches. S12 Fig. Phylogenetic trees inferred by Bayesian inference(BI) based on the 13 protein-coding genes and two rRNA genes (PCGs + rRNA). Bayesian posterior probabilities (BPPs) and bootstrap percentages (BP) are indicated. S13 Fig. Phylogenetic trees inferred by maximum likelihood (ML) based on the 13 protein-coding genes (PCGs). Bootstrap percentage (bp) is indicated on branches. S14 Fig. Phylogenetic trees inferred by maximum likelihood (ML) based on the 13 protein-coding genes and two rRNA genes (PCGs + rRNA). Bootstrap percentage (bp) is indicated on branches. S1 Table. Collection information for the 10 Cicadellidae species in this study. S2 Table. Summary statistics of the sequenced species. S3 Table. Sequence read archive accessions. S4 Table. Anagonalia emeiensis, Anagonalia melichari, Anatkina vespertinula, Erragonalia choui, Gunungidia aurantiifasciata, Kolla paulula, Nanatka castenea, Paratkina nigr [file pone.0329906.s001.zip › S11_Fig.tif]

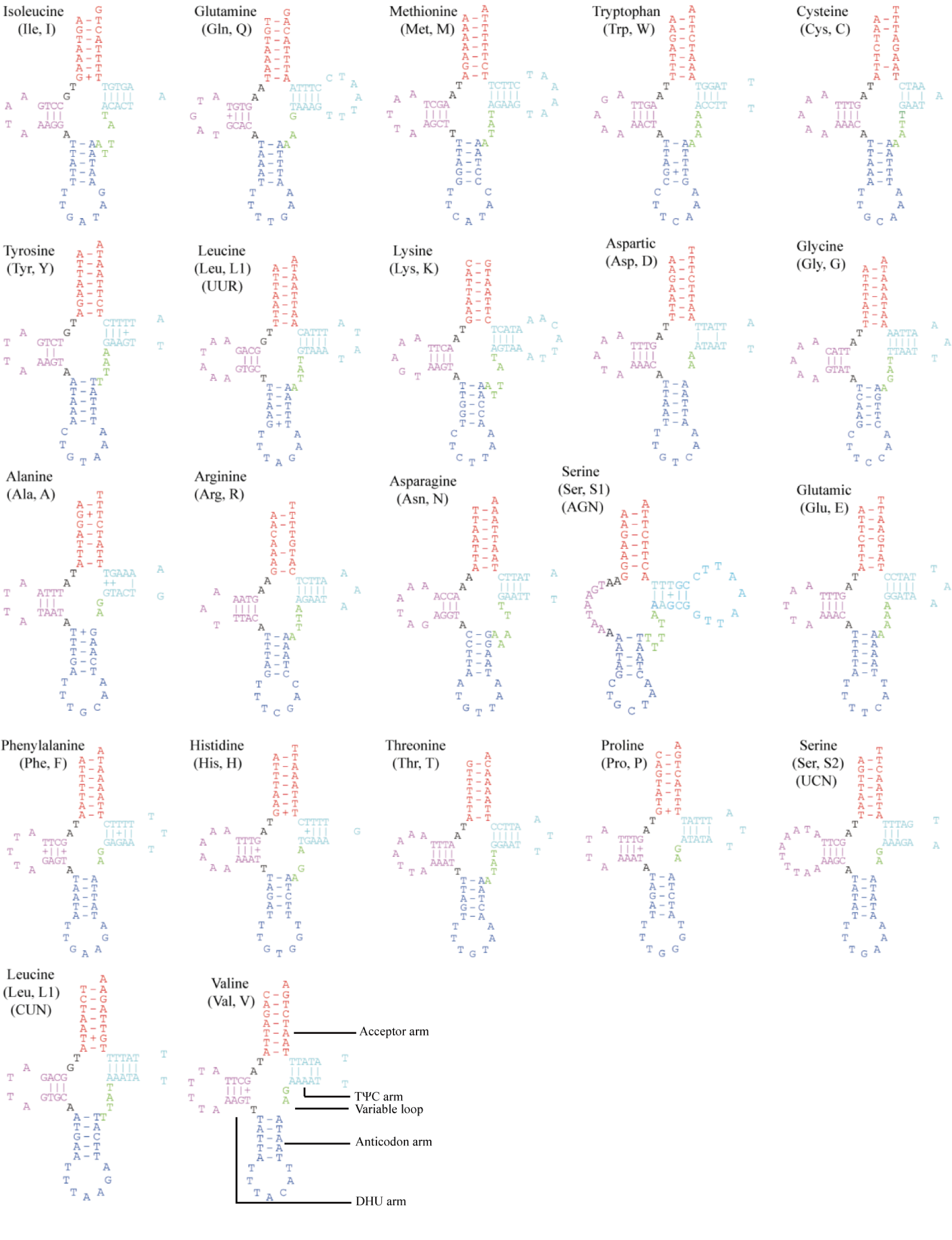

Supplement: S1 File — S1 Fig. Predicted secondary cloverleaf structure for the tRNAs of Anagonalia emeiensis. S2 Fig. Predicted secondary cloverleaf structure for the tRNAs of Anagonalia melichari. S3 Fig. Predicted secondary cloverleaf structure for the tRNAs of Anatkina vespertinula. S4 Fig. Predicted secondary cloverleaf structure for the tRNAs of Erragonalia choui. S5 Fig. Predicted secondary cloverleaf structure for the tRNAs of Gunungidia aurantiifasciata. S6 Fig. Predicted secondary cloverleaf structure for the tRNAs of kolla paulula. S7 Fig. Predicted secondary cloverleaf structure for the tRNAs of Nanatka castenea. S8 Fig. Predicted secondary cloverleaf structure for the tRNAs of Paratkina nigrifasciana. S9 Fig. Predicted secondary cloverleaf structure for the tRNAs of Seasogonia rosea. S10 Fig. Predicted secondary cloverleaf structure for the tRNAs of Stenatkina angustata. S11 Fig. Phylogenetic trees inferred by Bayesian inference (BI) based on the 13 protein-coding genes (PCGs). Bayesian posterior probabilities (BPPs) and bootstrap percentages (BP) are indicated on branches. S12 Fig. Phylogenetic trees inferred by Bayesian inference(BI) based on the 13 protein-coding genes and two rRNA genes (PCGs + rRNA). Bayesian posterior probabilities (BPPs) and bootstrap percentages (BP) are indicated. S13 Fig. Phylogenetic trees inferred by maximum likelihood (ML) based on the 13 protein-coding genes (PCGs). Bootstrap percentage (bp) is indicated on branches. S14 Fig. Phylogenetic trees inferred by maximum likelihood (ML) based on the 13 protein-coding genes and two rRNA genes (PCGs + rRNA). Bootstrap percentage (bp) is indicated on branches. S1 Table. Collection information for the 10 Cicadellidae species in this study. S2 Table. Summary statistics of the sequenced species. S3 Table. Sequence read archive accessions. S4 Table. Anagonalia emeiensis, Anagonalia melichari, Anatkina vespertinula, Erragonalia choui, Gunungidia aurantiifasciata, Kolla paulula, Nanatka castenea, Paratkina nigr [file pone.0329906.s001.zip › S10_Fig.tif]

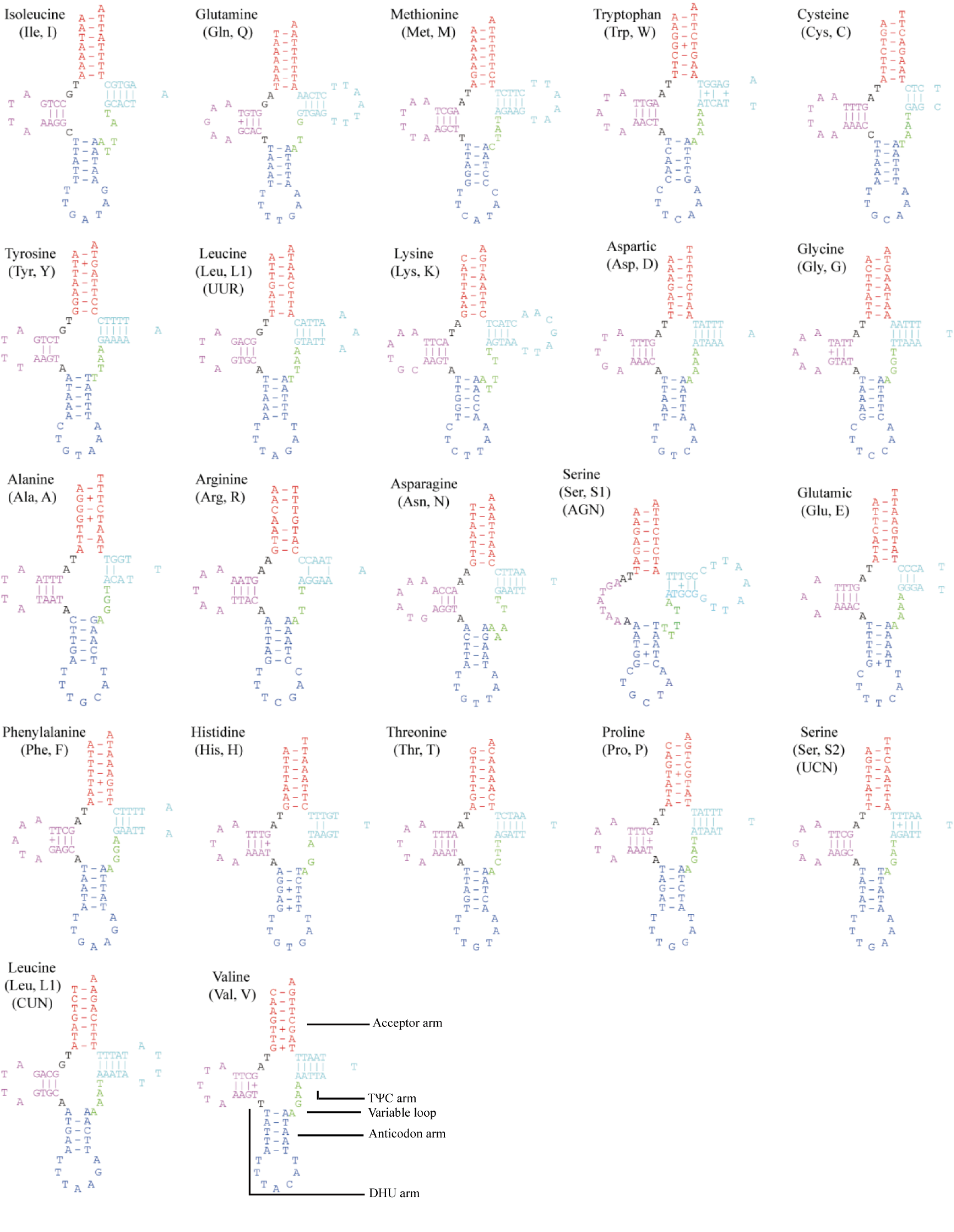

Supplement: S1 File — S1 Fig. Predicted secondary cloverleaf structure for the tRNAs of Anagonalia emeiensis. S2 Fig. Predicted secondary cloverleaf structure for the tRNAs of Anagonalia melichari. S3 Fig. Predicted secondary cloverleaf structure for the tRNAs of Anatkina vespertinula. S4 Fig. Predicted secondary cloverleaf structure for the tRNAs of Erragonalia choui. S5 Fig. Predicted secondary cloverleaf structure for the tRNAs of Gunungidia aurantiifasciata. S6 Fig. Predicted secondary cloverleaf structure for the tRNAs of kolla paulula. S7 Fig. Predicted secondary cloverleaf structure for the tRNAs of Nanatka castenea. S8 Fig. Predicted secondary cloverleaf structure for the tRNAs of Paratkina nigrifasciana. S9 Fig. Predicted secondary cloverleaf structure for the tRNAs of Seasogonia rosea. S10 Fig. Predicted secondary cloverleaf structure for the tRNAs of Stenatkina angustata. S11 Fig. Phylogenetic trees inferred by Bayesian inference (BI) based on the 13 protein-coding genes (PCGs). Bayesian posterior probabilities (BPPs) and bootstrap percentages (BP) are indicated on branches. S12 Fig. Phylogenetic trees inferred by Bayesian inference(BI) based on the 13 protein-coding genes and two rRNA genes (PCGs + rRNA). Bayesian posterior probabilities (BPPs) and bootstrap percentages (BP) are indicated. S13 Fig. Phylogenetic trees inferred by maximum likelihood (ML) based on the 13 protein-coding genes (PCGs). Bootstrap percentage (bp) is indicated on branches. S14 Fig. Phylogenetic trees inferred by maximum likelihood (ML) based on the 13 protein-coding genes and two rRNA genes (PCGs + rRNA). Bootstrap percentage (bp) is indicated on branches. S1 Table. Collection information for the 10 Cicadellidae species in this study. S2 Table. Summary statistics of the sequenced species. S3 Table. Sequence read archive accessions. S4 Table. Anagonalia emeiensis, Anagonalia melichari, Anatkina vespertinula, Erragonalia choui, Gunungidia aurantiifasciata, Kolla paulula, Nanatka castenea, Paratkina nigr [file pone.0329906.s001.zip › S9_Fig.tif]

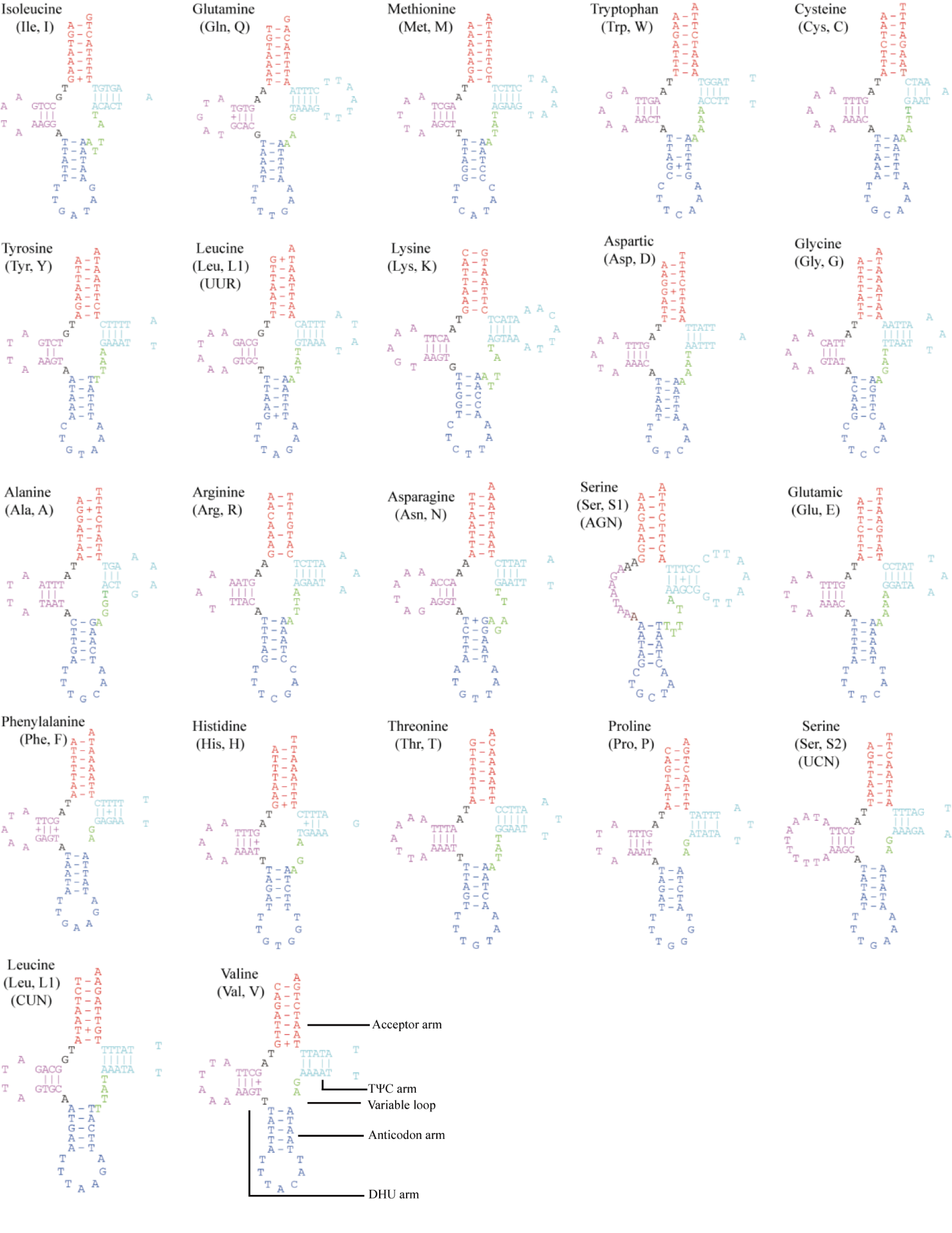

Supplement: S1 File — S1 Fig. Predicted secondary cloverleaf structure for the tRNAs of Anagonalia emeiensis. S2 Fig. Predicted secondary cloverleaf structure for the tRNAs of Anagonalia melichari. S3 Fig. Predicted secondary cloverleaf structure for the tRNAs of Anatkina vespertinula. S4 Fig. Predicted secondary cloverleaf structure for the tRNAs of Erragonalia choui. S5 Fig. Predicted secondary cloverleaf structure for the tRNAs of Gunungidia aurantiifasciata. S6 Fig. Predicted secondary cloverleaf structure for the tRNAs of kolla paulula. S7 Fig. Predicted secondary cloverleaf structure for the tRNAs of Nanatka castenea. S8 Fig. Predicted secondary cloverleaf structure for the tRNAs of Paratkina nigrifasciana. S9 Fig. Predicted secondary cloverleaf structure for the tRNAs of Seasogonia rosea. S10 Fig. Predicted secondary cloverleaf structure for the tRNAs of Stenatkina angustata. S11 Fig. Phylogenetic trees inferred by Bayesian inference (BI) based on the 13 protein-coding genes (PCGs). Bayesian posterior probabilities (BPPs) and bootstrap percentages (BP) are indicated on branches. S12 Fig. Phylogenetic trees inferred by Bayesian inference(BI) based on the 13 protein-coding genes and two rRNA genes (PCGs + rRNA). Bayesian posterior probabilities (BPPs) and bootstrap percentages (BP) are indicated. S13 Fig. Phylogenetic trees inferred by maximum likelihood (ML) based on the 13 protein-coding genes (PCGs). Bootstrap percentage (bp) is indicated on branches. S14 Fig. Phylogenetic trees inferred by maximum likelihood (ML) based on the 13 protein-coding genes and two rRNA genes (PCGs + rRNA). Bootstrap percentage (bp) is indicated on branches. S1 Table. Collection information for the 10 Cicadellidae species in this study. S2 Table. Summary statistics of the sequenced species. S3 Table. Sequence read archive accessions. S4 Table. Anagonalia emeiensis, Anagonalia melichari, Anatkina vespertinula, Erragonalia choui, Gunungidia aurantiifasciata, Kolla paulula, Nanatka castenea, Paratkina nigr [file pone.0329906.s001.zip › S8_Fig.tif]

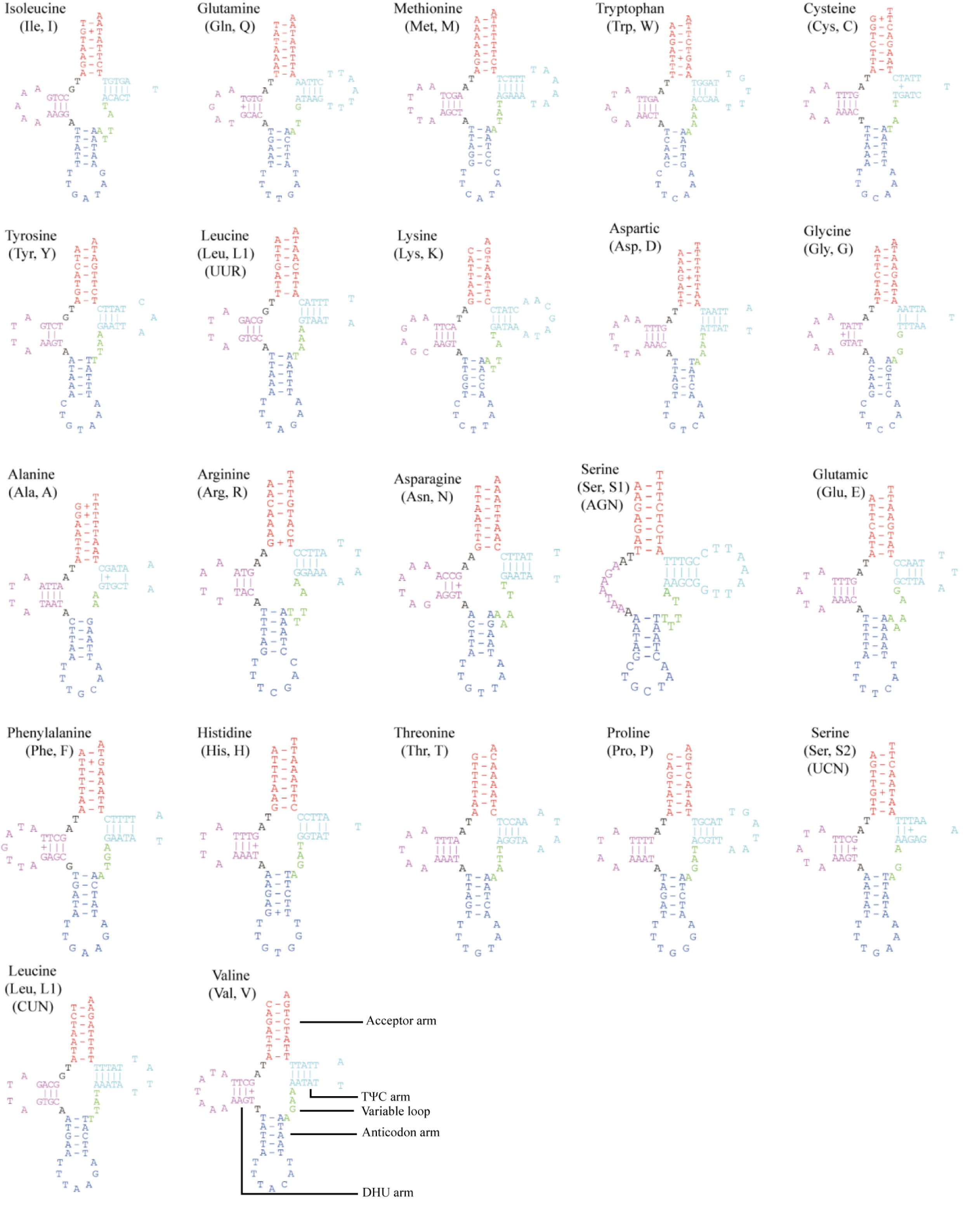

Supplement: S1 File — S1 Fig. Predicted secondary cloverleaf structure for the tRNAs of Anagonalia emeiensis. S2 Fig. Predicted secondary cloverleaf structure for the tRNAs of Anagonalia melichari. S3 Fig. Predicted secondary cloverleaf structure for the tRNAs of Anatkina vespertinula. S4 Fig. Predicted secondary cloverleaf structure for the tRNAs of Erragonalia choui. S5 Fig. Predicted secondary cloverleaf structure for the tRNAs of Gunungidia aurantiifasciata. S6 Fig. Predicted secondary cloverleaf structure for the tRNAs of kolla paulula. S7 Fig. Predicted secondary cloverleaf structure for the tRNAs of Nanatka castenea. S8 Fig. Predicted secondary cloverleaf structure for the tRNAs of Paratkina nigrifasciana. S9 Fig. Predicted secondary cloverleaf structure for the tRNAs of Seasogonia rosea. S10 Fig. Predicted secondary cloverleaf structure for the tRNAs of Stenatkina angustata. S11 Fig. Phylogenetic trees inferred by Bayesian inference (BI) based on the 13 protein-coding genes (PCGs). Bayesian posterior probabilities (BPPs) and bootstrap percentages (BP) are indicated on branches. S12 Fig. Phylogenetic trees inferred by Bayesian inference(BI) based on the 13 protein-coding genes and two rRNA genes (PCGs + rRNA). Bayesian posterior probabilities (BPPs) and bootstrap percentages (BP) are indicated. S13 Fig. Phylogenetic trees inferred by maximum likelihood (ML) based on the 13 protein-coding genes (PCGs). Bootstrap percentage (bp) is indicated on branches. S14 Fig. Phylogenetic trees inferred by maximum likelihood (ML) based on the 13 protein-coding genes and two rRNA genes (PCGs + rRNA). Bootstrap percentage (bp) is indicated on branches. S1 Table. Collection information for the 10 Cicadellidae species in this study. S2 Table. Summary statistics of the sequenced species. S3 Table. Sequence read archive accessions. S4 Table. Anagonalia emeiensis, Anagonalia melichari, Anatkina vespertinula, Erragonalia choui, Gunungidia aurantiifasciata, Kolla paulula, Nanatka castenea, Paratkina nigr [file pone.0329906.s001.zip › S7_Fig.tif]

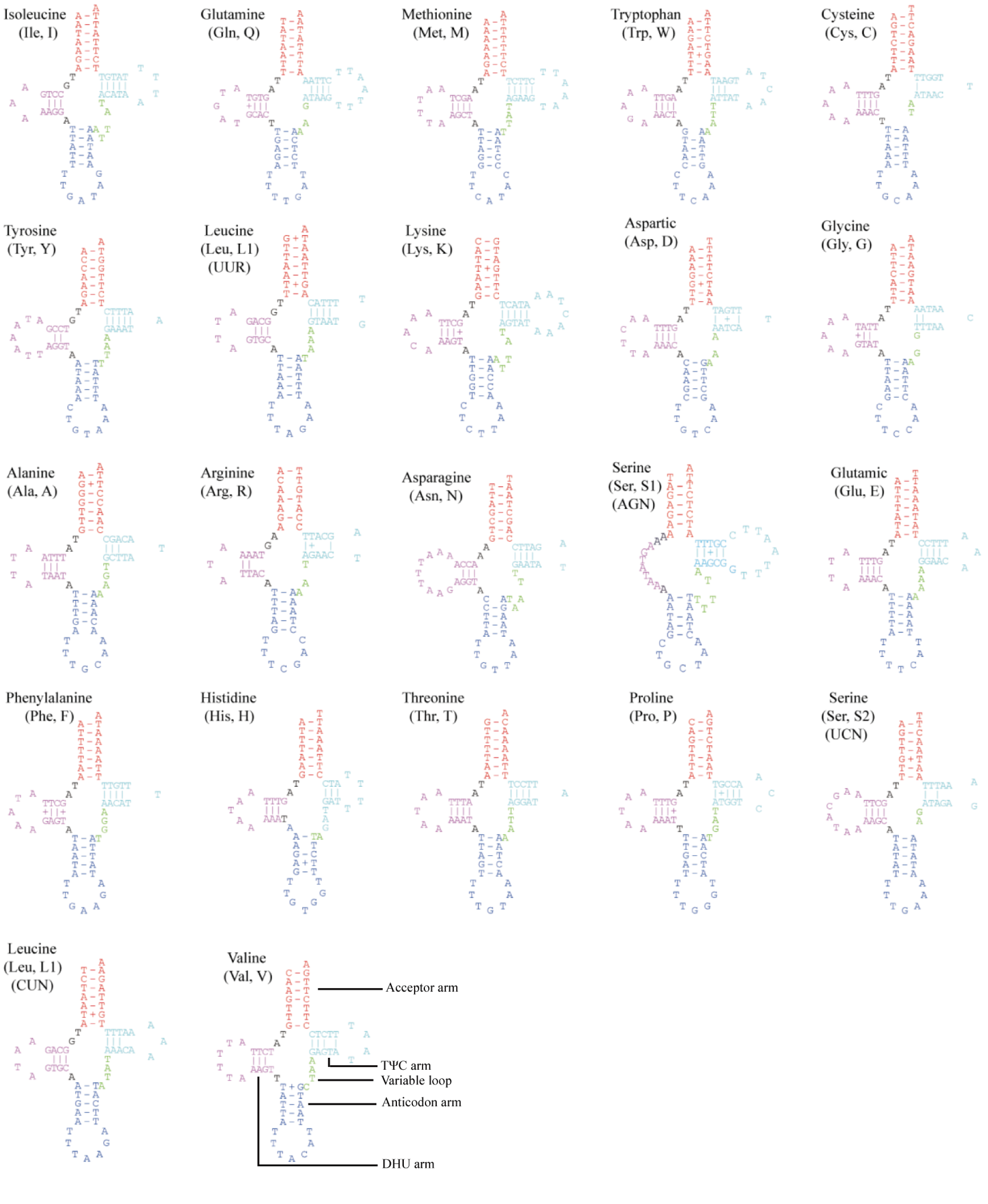

Supplement: S1 File — S1 Fig. Predicted secondary cloverleaf structure for the tRNAs of Anagonalia emeiensis. S2 Fig. Predicted secondary cloverleaf structure for the tRNAs of Anagonalia melichari. S3 Fig. Predicted secondary cloverleaf structure for the tRNAs of Anatkina vespertinula. S4 Fig. Predicted secondary cloverleaf structure for the tRNAs of Erragonalia choui. S5 Fig. Predicted secondary cloverleaf structure for the tRNAs of Gunungidia aurantiifasciata. S6 Fig. Predicted secondary cloverleaf structure for the tRNAs of kolla paulula. S7 Fig. Predicted secondary cloverleaf structure for the tRNAs of Nanatka castenea. S8 Fig. Predicted secondary cloverleaf structure for the tRNAs of Paratkina nigrifasciana. S9 Fig. Predicted secondary cloverleaf structure for the tRNAs of Seasogonia rosea. S10 Fig. Predicted secondary cloverleaf structure for the tRNAs of Stenatkina angustata. S11 Fig. Phylogenetic trees inferred by Bayesian inference (BI) based on the 13 protein-coding genes (PCGs). Bayesian posterior probabilities (BPPs) and bootstrap percentages (BP) are indicated on branches. S12 Fig. Phylogenetic trees inferred by Bayesian inference(BI) based on the 13 protein-coding genes and two rRNA genes (PCGs + rRNA). Bayesian posterior probabilities (BPPs) and bootstrap percentages (BP) are indicated. S13 Fig. Phylogenetic trees inferred by maximum likelihood (ML) based on the 13 protein-coding genes (PCGs). Bootstrap percentage (bp) is indicated on branches. S14 Fig. Phylogenetic trees inferred by maximum likelihood (ML) based on the 13 protein-coding genes and two rRNA genes (PCGs + rRNA). Bootstrap percentage (bp) is indicated on branches. S1 Table. Collection information for the 10 Cicadellidae species in this study. S2 Table. Summary statistics of the sequenced species. S3 Table. Sequence read archive accessions. S4 Table. Anagonalia emeiensis, Anagonalia melichari, Anatkina vespertinula, Erragonalia choui, Gunungidia aurantiifasciata, Kolla paulula, Nanatka castenea, Paratkina nigr [file pone.0329906.s001.zip › S6_Fig.tif]

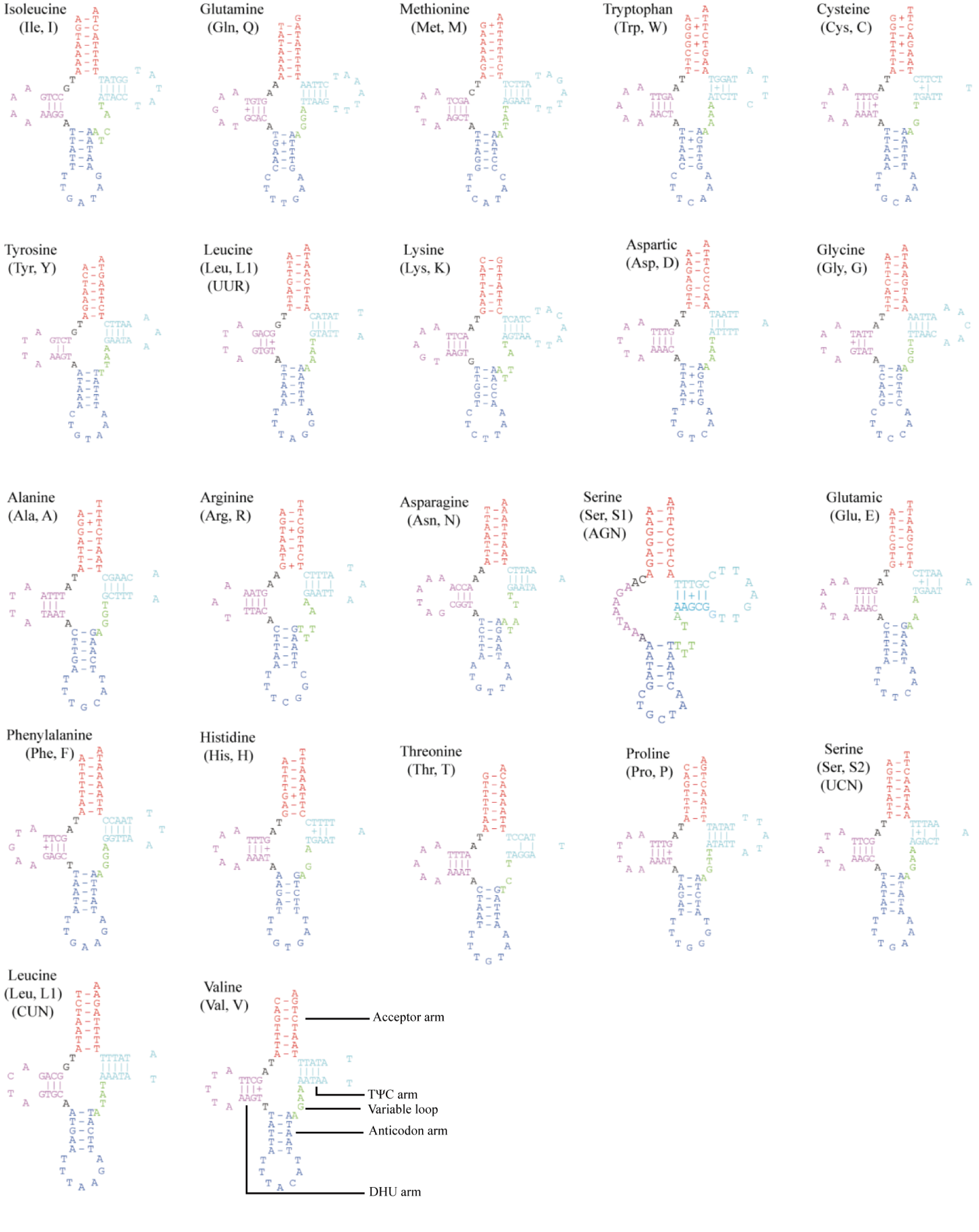

Supplement: S1 File — S1 Fig. Predicted secondary cloverleaf structure for the tRNAs of Anagonalia emeiensis. S2 Fig. Predicted secondary cloverleaf structure for the tRNAs of Anagonalia melichari. S3 Fig. Predicted secondary cloverleaf structure for the tRNAs of Anatkina vespertinula. S4 Fig. Predicted secondary cloverleaf structure for the tRNAs of Erragonalia choui. S5 Fig. Predicted secondary cloverleaf structure for the tRNAs of Gunungidia aurantiifasciata. S6 Fig. Predicted secondary cloverleaf structure for the tRNAs of kolla paulula. S7 Fig. Predicted secondary cloverleaf structure for the tRNAs of Nanatka castenea. S8 Fig. Predicted secondary cloverleaf structure for the tRNAs of Paratkina nigrifasciana. S9 Fig. Predicted secondary cloverleaf structure for the tRNAs of Seasogonia rosea. S10 Fig. Predicted secondary cloverleaf structure for the tRNAs of Stenatkina angustata. S11 Fig. Phylogenetic trees inferred by Bayesian inference (BI) based on the 13 protein-coding genes (PCGs). Bayesian posterior probabilities (BPPs) and bootstrap percentages (BP) are indicated on branches. S12 Fig. Phylogenetic trees inferred by Bayesian inference(BI) based on the 13 protein-coding genes and two rRNA genes (PCGs + rRNA). Bayesian posterior probabilities (BPPs) and bootstrap percentages (BP) are indicated. S13 Fig. Phylogenetic trees inferred by maximum likelihood (ML) based on the 13 protein-coding genes (PCGs). Bootstrap percentage (bp) is indicated on branches. S14 Fig. Phylogenetic trees inferred by maximum likelihood (ML) based on the 13 protein-coding genes and two rRNA genes (PCGs + rRNA). Bootstrap percentage (bp) is indicated on branches. S1 Table. Collection information for the 10 Cicadellidae species in this study. S2 Table. Summary statistics of the sequenced species. S3 Table. Sequence read archive accessions. S4 Table. Anagonalia emeiensis, Anagonalia melichari, Anatkina vespertinula, Erragonalia choui, Gunungidia aurantiifasciata, Kolla paulula, Nanatka castenea, Paratkina nigr [file pone.0329906.s001.zip › S5_Fig.tif]

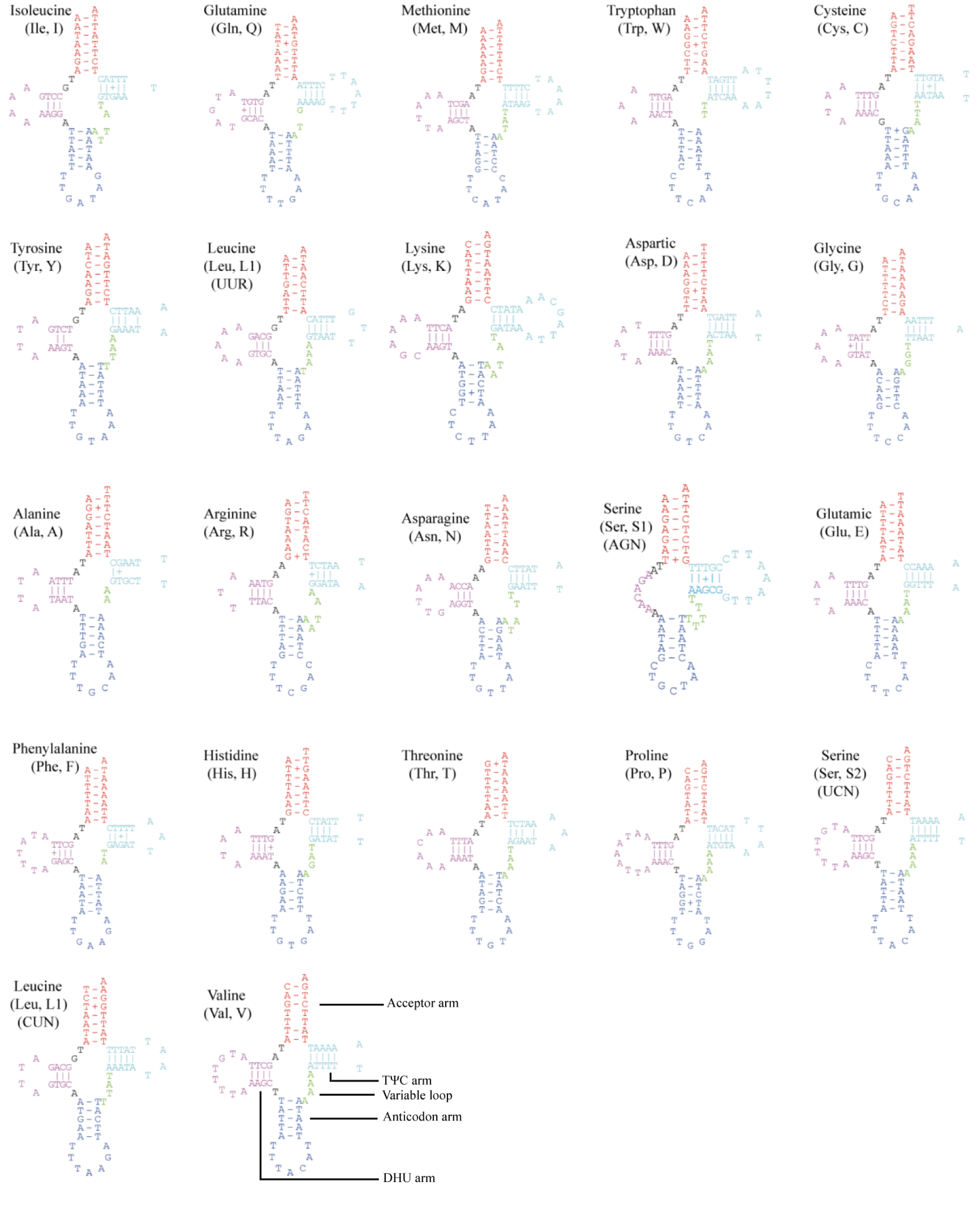

Supplement: S1 File — S1 Fig. Predicted secondary cloverleaf structure for the tRNAs of Anagonalia emeiensis. S2 Fig. Predicted secondary cloverleaf structure for the tRNAs of Anagonalia melichari. S3 Fig. Predicted secondary cloverleaf structure for the tRNAs of Anatkina vespertinula. S4 Fig. Predicted secondary cloverleaf structure for the tRNAs of Erragonalia choui. S5 Fig. Predicted secondary cloverleaf structure for the tRNAs of Gunungidia aurantiifasciata. S6 Fig. Predicted secondary cloverleaf structure for the tRNAs of kolla paulula. S7 Fig. Predicted secondary cloverleaf structure for the tRNAs of Nanatka castenea. S8 Fig. Predicted secondary cloverleaf structure for the tRNAs of Paratkina nigrifasciana. S9 Fig. Predicted secondary cloverleaf structure for the tRNAs of Seasogonia rosea. S10 Fig. Predicted secondary cloverleaf structure for the tRNAs of Stenatkina angustata. S11 Fig. Phylogenetic trees inferred by Bayesian inference (BI) based on the 13 protein-coding genes (PCGs). Bayesian posterior probabilities (BPPs) and bootstrap percentages (BP) are indicated on branches. S12 Fig. Phylogenetic trees inferred by Bayesian inference(BI) based on the 13 protein-coding genes and two rRNA genes (PCGs + rRNA). Bayesian posterior probabilities (BPPs) and bootstrap percentages (BP) are indicated. S13 Fig. Phylogenetic trees inferred by maximum likelihood (ML) based on the 13 protein-coding genes (PCGs). Bootstrap percentage (bp) is indicated on branches. S14 Fig. Phylogenetic trees inferred by maximum likelihood (ML) based on the 13 protein-coding genes and two rRNA genes (PCGs + rRNA). Bootstrap percentage (bp) is indicated on branches. S1 Table. Collection information for the 10 Cicadellidae species in this study. S2 Table. Summary statistics of the sequenced species. S3 Table. Sequence read archive accessions. S4 Table. Anagonalia emeiensis, Anagonalia melichari, Anatkina vespertinula, Erragonalia choui, Gunungidia aurantiifasciata, Kolla paulula, Nanatka castenea, Paratkina nigr [file pone.0329906.s001.zip › S4_Fig.tif]

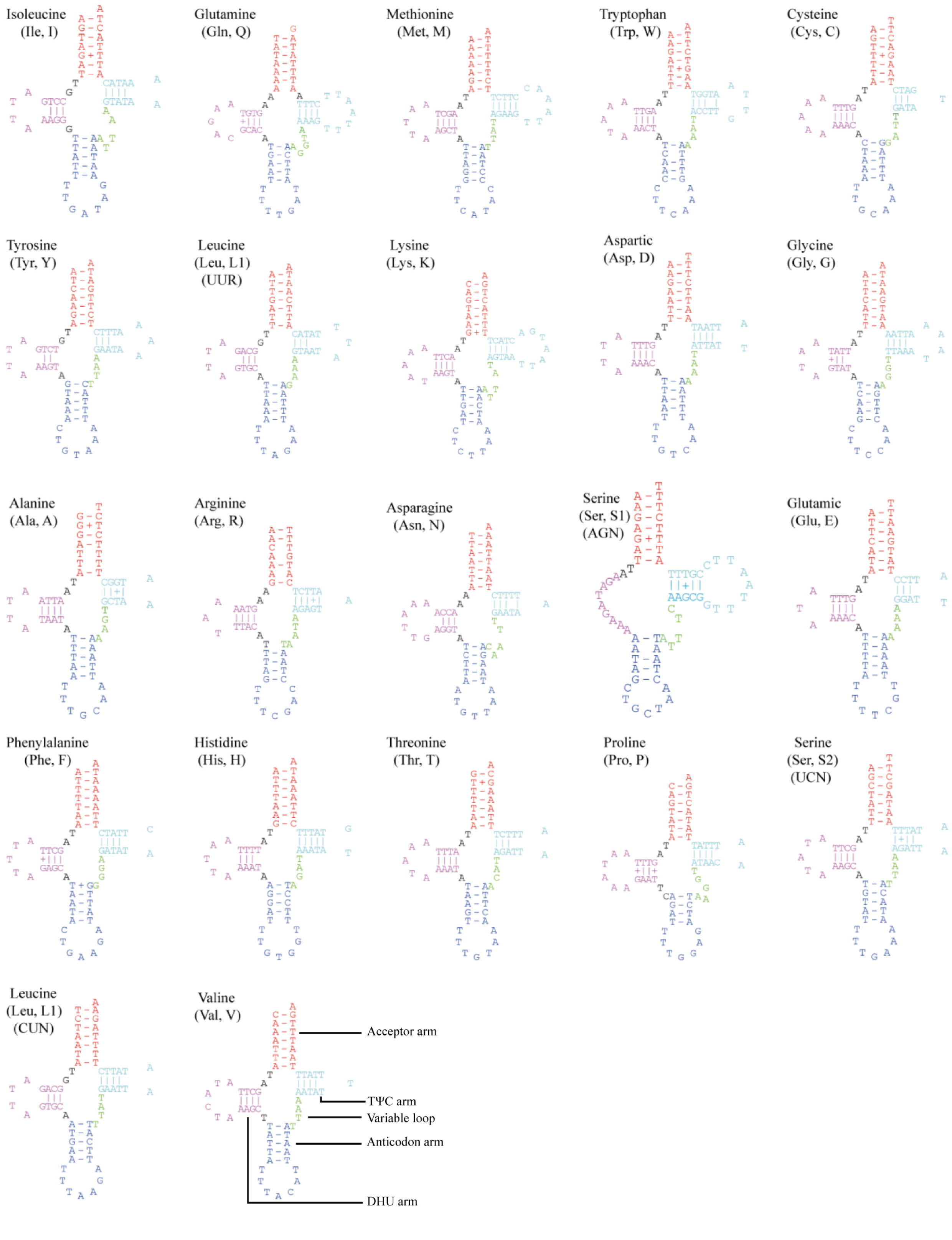

Supplement: S1 File — S1 Fig. Predicted secondary cloverleaf structure for the tRNAs of Anagonalia emeiensis. S2 Fig. Predicted secondary cloverleaf structure for the tRNAs of Anagonalia melichari. S3 Fig. Predicted secondary cloverleaf structure for the tRNAs of Anatkina vespertinula. S4 Fig. Predicted secondary cloverleaf structure for the tRNAs of Erragonalia choui. S5 Fig. Predicted secondary cloverleaf structure for the tRNAs of Gunungidia aurantiifasciata. S6 Fig. Predicted secondary cloverleaf structure for the tRNAs of kolla paulula. S7 Fig. Predicted secondary cloverleaf structure for the tRNAs of Nanatka castenea. S8 Fig. Predicted secondary cloverleaf structure for the tRNAs of Paratkina nigrifasciana. S9 Fig. Predicted secondary cloverleaf structure for the tRNAs of Seasogonia rosea. S10 Fig. Predicted secondary cloverleaf structure for the tRNAs of Stenatkina angustata. S11 Fig. Phylogenetic trees inferred by Bayesian inference (BI) based on the 13 protein-coding genes (PCGs). Bayesian posterior probabilities (BPPs) and bootstrap percentages (BP) are indicated on branches. S12 Fig. Phylogenetic trees inferred by Bayesian inference(BI) based on the 13 protein-coding genes and two rRNA genes (PCGs + rRNA). Bayesian posterior probabilities (BPPs) and bootstrap percentages (BP) are indicated. S13 Fig. Phylogenetic trees inferred by maximum likelihood (ML) based on the 13 protein-coding genes (PCGs). Bootstrap percentage (bp) is indicated on branches. S14 Fig. Phylogenetic trees inferred by maximum likelihood (ML) based on the 13 protein-coding genes and two rRNA genes (PCGs + rRNA). Bootstrap percentage (bp) is indicated on branches. S1 Table. Collection information for the 10 Cicadellidae species in this study. S2 Table. Summary statistics of the sequenced species. S3 Table. Sequence read archive accessions. S4 Table. Anagonalia emeiensis, Anagonalia melichari, Anatkina vespertinula, Erragonalia choui, Gunungidia aurantiifasciata, Kolla paulula, Nanatka castenea, Paratkina nigr [file pone.0329906.s001.zip › S3_Fig.tif]

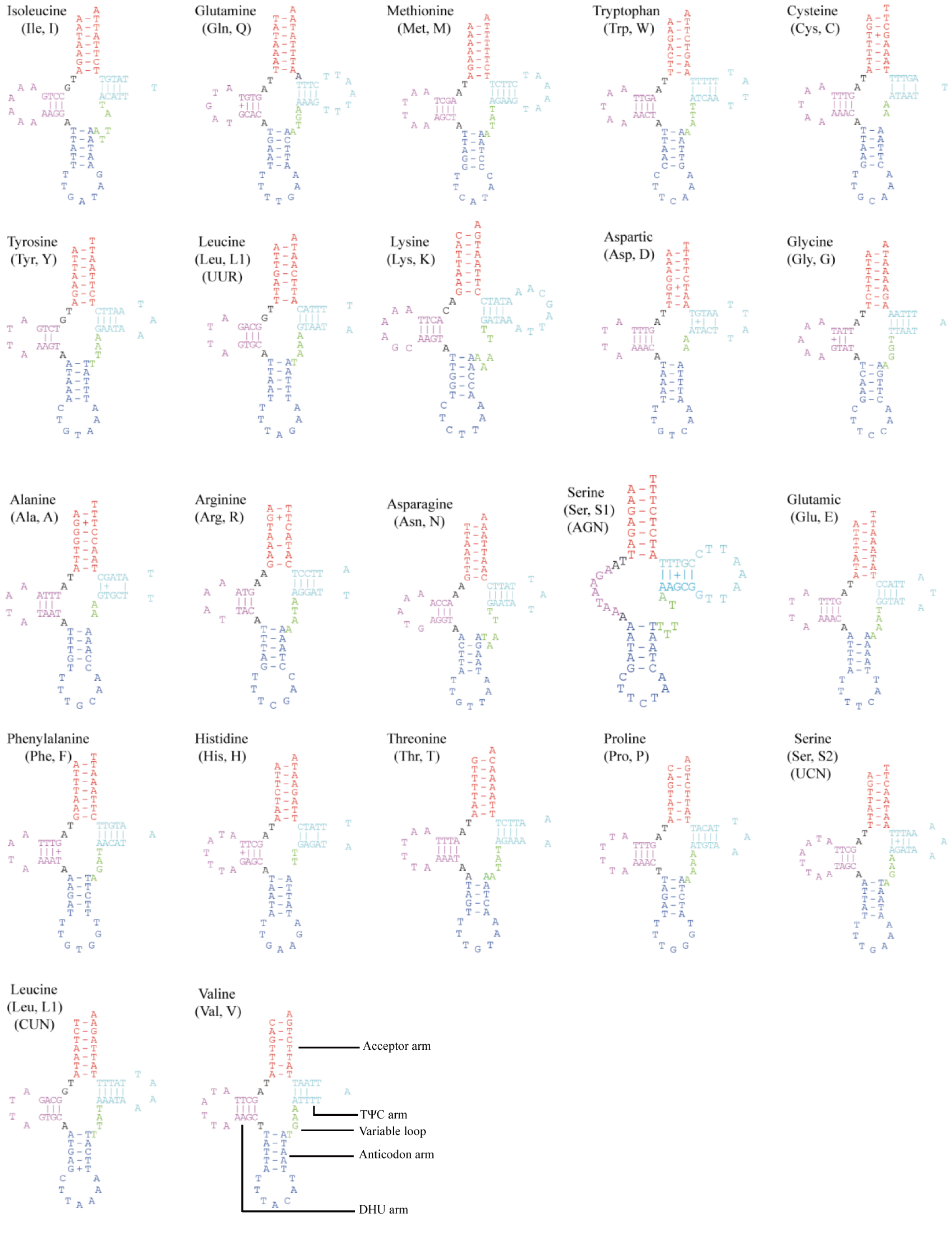

Supplement: S1 File — S1 Fig. Predicted secondary cloverleaf structure for the tRNAs of Anagonalia emeiensis. S2 Fig. Predicted secondary cloverleaf structure for the tRNAs of Anagonalia melichari. S3 Fig. Predicted secondary cloverleaf structure for the tRNAs of Anatkina vespertinula. S4 Fig. Predicted secondary cloverleaf structure for the tRNAs of Erragonalia choui. S5 Fig. Predicted secondary cloverleaf structure for the tRNAs of Gunungidia aurantiifasciata. S6 Fig. Predicted secondary cloverleaf structure for the tRNAs of kolla paulula. S7 Fig. Predicted secondary cloverleaf structure for the tRNAs of Nanatka castenea. S8 Fig. Predicted secondary cloverleaf structure for the tRNAs of Paratkina nigrifasciana. S9 Fig. Predicted secondary cloverleaf structure for the tRNAs of Seasogonia rosea. S10 Fig. Predicted secondary cloverleaf structure for the tRNAs of Stenatkina angustata. S11 Fig. Phylogenetic trees inferred by Bayesian inference (BI) based on the 13 protein-coding genes (PCGs). Bayesian posterior probabilities (BPPs) and bootstrap percentages (BP) are indicated on branches. S12 Fig. Phylogenetic trees inferred by Bayesian inference(BI) based on the 13 protein-coding genes and two rRNA genes (PCGs + rRNA). Bayesian posterior probabilities (BPPs) and bootstrap percentages (BP) are indicated. S13 Fig. Phylogenetic trees inferred by maximum likelihood (ML) based on the 13 protein-coding genes (PCGs). Bootstrap percentage (bp) is indicated on branches. S14 Fig. Phylogenetic trees inferred by maximum likelihood (ML) based on the 13 protein-coding genes and two rRNA genes (PCGs + rRNA). Bootstrap percentage (bp) is indicated on branches. S1 Table. Collection information for the 10 Cicadellidae species in this study. S2 Table. Summary statistics of the sequenced species. S3 Table. Sequence read archive accessions. S4 Table. Anagonalia emeiensis, Anagonalia melichari, Anatkina vespertinula, Erragonalia choui, Gunungidia aurantiifasciata, Kolla paulula, Nanatka castenea, Paratkina nigr [file pone.0329906.s001.zip › S2_Fig.tif]

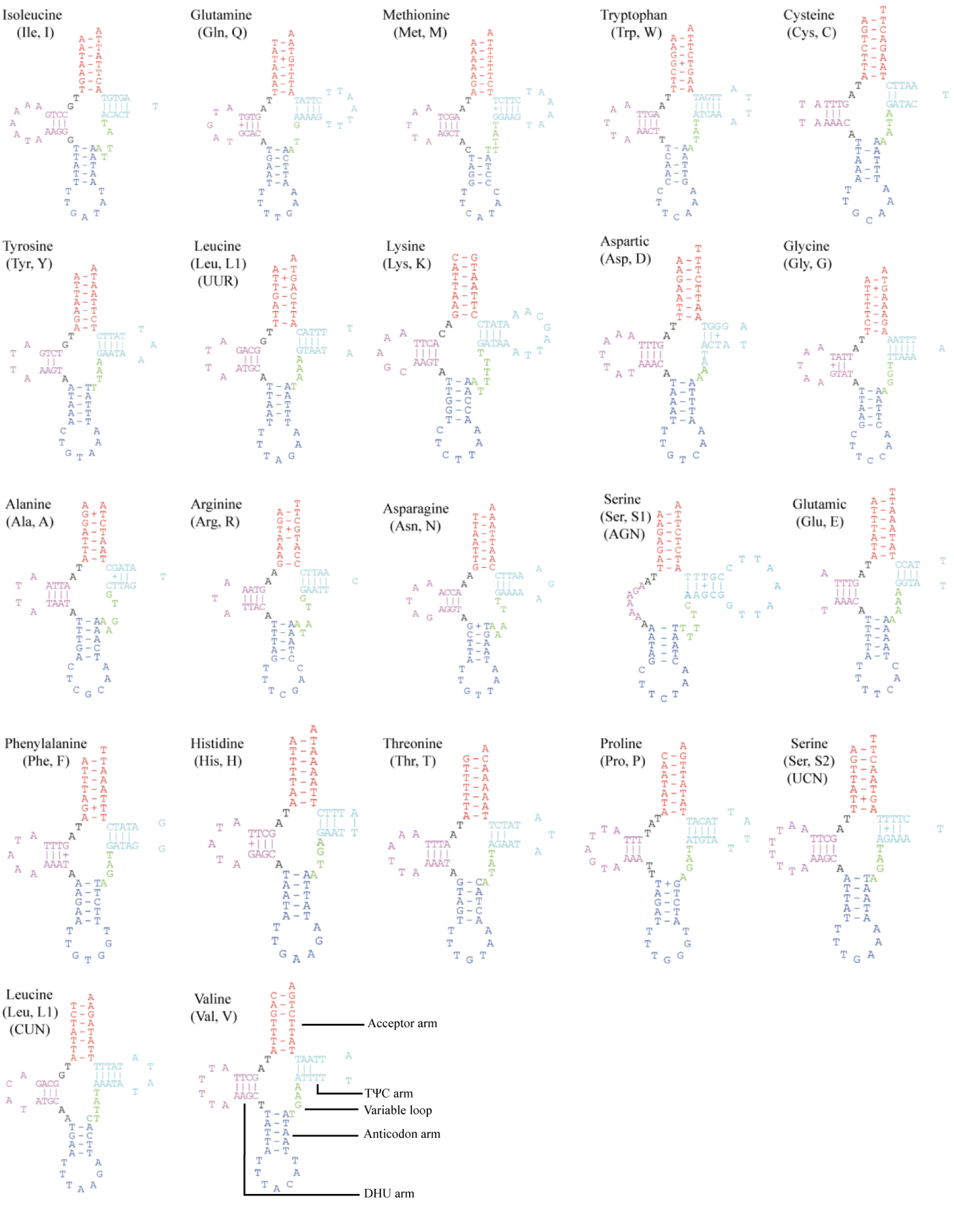

Supplement: S1 File — S1 Fig. Predicted secondary cloverleaf structure for the tRNAs of Anagonalia emeiensis. S2 Fig. Predicted secondary cloverleaf structure for the tRNAs of Anagonalia melichari. S3 Fig. Predicted secondary cloverleaf structure for the tRNAs of Anatkina vespertinula. S4 Fig. Predicted secondary cloverleaf structure for the tRNAs of Erragonalia choui. S5 Fig. Predicted secondary cloverleaf structure for the tRNAs of Gunungidia aurantiifasciata. S6 Fig. Predicted secondary cloverleaf structure for the tRNAs of kolla paulula. S7 Fig. Predicted secondary cloverleaf structure for the tRNAs of Nanatka castenea. S8 Fig. Predicted secondary cloverleaf structure for the tRNAs of Paratkina nigrifasciana. S9 Fig. Predicted secondary cloverleaf structure for the tRNAs of Seasogonia rosea. S10 Fig. Predicted secondary cloverleaf structure for the tRNAs of Stenatkina angustata. S11 Fig. Phylogenetic trees inferred by Bayesian inference (BI) based on the 13 protein-coding genes (PCGs). Bayesian posterior probabilities (BPPs) and bootstrap percentages (BP) are indicated on branches. S12 Fig. Phylogenetic trees inferred by Bayesian inference(BI) based on the 13 protein-coding genes and two rRNA genes (PCGs + rRNA). Bayesian posterior probabilities (BPPs) and bootstrap percentages (BP) are indicated. S13 Fig. Phylogenetic trees inferred by maximum likelihood (ML) based on the 13 protein-coding genes (PCGs). Bootstrap percentage (bp) is indicated on branches. S14 Fig. Phylogenetic trees inferred by maximum likelihood (ML) based on the 13 protein-coding genes and two rRNA genes (PCGs + rRNA). Bootstrap percentage (bp) is indicated on branches. S1 Table. Collection information for the 10 Cicadellidae species in this study. S2 Table. Summary statistics of the sequenced species. S3 Table. Sequence read archive accessions. S4 Table. Anagonalia emeiensis, Anagonalia melichari, Anatkina vespertinula, Erragonalia choui, Gunungidia aurantiifasciata, Kolla paulula, Nanatka castenea, Paratkina nigr [file pone.0329906.s001.zip › S1_Fig.tif]
