# Supplementary material for: Phylogenetic investigation and mitochondrial genome description of ten species in nine genera of Cicadellinae from China (Hemiptera: Cicadellidae)
Source: PLoS One. 2025 Aug 12;20(8):e0329906. doi: 10.1371/journal.pone.0329906 (PMC12342267; doi:10.1371/journal.pone.0329906)
Supplement: Attachment — Submitted filename: Response to Reviewers.docx [file pone.0329906.s002.docx]

Dear Reviewer,

Greetings from all authors!

We express our gratitude for your meticulous perusal, helpful comments, and constructive suggestions, which have substantially enhanced the exposition of our manuscript. We have carefully considered all comments and revised our manuscript accordingly, which includes writing polish, data availability, etc. For more details about our revised work, kindly refer to the response provided below.

**Reviewer 1**

**Query:** The authors may provide a supplementary table with summary statistics of the sequenced species, for example, number of sequenced raw reads, number of clean reads, number of reads used to assemble the mitochondrial genomes, mapping information, such as coverage and depth of the assembled genomes.

**Response:** Thanks for your suggestions. Table has been supplemented (Table S2).

**Q:** The raw reads must be deposited to a public repository (for example, SRA) and made available.

**Response:** Regards for your recommendations. Raw data has been uploaded to the Sequence Read Archive (Table S3).

**Q:** There is limited explanation of the methodology used for phylogenetic analyses. On Materials and Methods, section 2.3 Phylogenetic analyses, the authors stated that “the best partitioning scheme and evolutionary models for 13 predefined partitions were selected using PartitionFinder2”, however the IQ-TREE (ML analysis) was used on ‘Auto’ mode for model selection. If IQ-TREE automatically selected the model, it is unclear why the best partition scheme and model were selected with PartitionFinder2?

**Response:** Thank for your comment. IQ-TREE (ML analysis) was used on 'Auto' mode for model selection in order to identify PartitionFinder2 selected partitions and models automatically (Table S8). The description in the text has been changed (Line number: 120).

**Q:** The authors claimed that all ten species of Cicadellinae had their whole mitochondrial genomes assembled, however on Fig. 2 there is no annotation of ‘Control Region’ for two species (*Anagonalia emeiesis* and *Seasogonia rosea*). Please correct the figure or provide clarification on the annotation process.

**Response:** Thank you for your comments, (*Anagonalia emeiesis* and *Seasogonia rosea*) the control area is incomplete and we have used Geneious for mapping and have never been able to complete the identification of the full control area, but its completeness does not affect the phylogenetic study, and the terminology has now been changed for the 10 species of COMPLETE (Line number: 145, 259).

**Q:** The authors stated at the introduction that the “leafhoppers are effective vectors of plant pathogens such as phytoplasmas, as well as other bacteria and viruses that cause illness in a variety of plants”. The MS lacks detailed discussion on the implications of the findings for broader evolutionary and ecological contexts. In the conclusion section, there is a lot of repeated information from the results.

**Response:** Thank you for your valuable comments. Leafhoppers cause direct feeding damage to plants and transmit plant pathogens. Some leafhopper species are agricultural pests, but this was not the focus of the Cicadelliane phylogenetic study in this paper, so they are not discussed in their ecological context. Duplicate information in the conclusion has been changed.

**Q:** The authors should improve figure captions with more detailed information about the figures themselves and methods employed to generate them. For example, what represents the inner circles of Fig. 1?

**Response:** Thanks for your comments. Genes are depicted as blocks of different colors. Genes on the J-strand are indicated by color blocks outside the circle; genes on the N-strand are indicated by color blocks inside the circle. Each circle is coloured in a different way to represent different information within the mitochondrial genome, such as the location and orientation of genes, the amount of GC, etc. . For example, the inner circle of Figure 1 represents the size range of the mitochondrial genome (Line number: 147).

**Reviewer 2**

**Query**: The results presented in this work are robust and well-described. However, to ensure the reproducibility of the study, it is crucial to include detailed information about the library preparation kits and sequencing method used, as well as the sequencing depth (number of sequenced reads) for each sample. I recommend that this information be provided as supplementary material, which will significantly enhance the contribution of your article to the scientific community.

**Response:** Thanks for your suggestions. The library preparation kits and sequencing methods used are described in Materials and Methods, and the kits were used according to the instructions, as well as the depth of sequencing (number of sequencing reads) for each sample, which is provided as Supplementary Material (Table S2).

We look forward to hearing from you at your earliest convenience.

Sincerely,

Likun Zhong, Maofa Yang
